# High Growing Temperature Changes Nutritional Value of Broccoli (*Brassica oleracea* L. convar. *botrytis* (L.) Alef. var. *cymosa* Duch.) Seedlings

**DOI:** 10.3390/foods12030582

**Published:** 2023-01-29

**Authors:** Daria Gmižić, Marija Pinterić, Maja Lazarus, Ivana Šola

**Affiliations:** 1 Department of Biology, Faculty of Science, University of Zagreb, Horvatovac 102a, 10000 Zagreb, Croatia; 2 Division of Molecular Medicine, Ruđer Bošković Institute, Bijenička 54, 10000 Zagreb, Croatia; 3 Analytical Toxicology and Mineral Metabolism Unit, Institute for Medical Research and Occupational Health, Ksaverska cesta 2, 10000 Zagreb, Croatia

**Keywords:** antioxidant capacity, Brassicaceae, climate change, food quality, global warming, in vitro cytotoxicity, macro- and microelements, phytochemical composition

## Abstract

High temperature (HT) causes physiological and biochemical changes in plants, which may influence their nutritional potential. This study aimed to evaluate the nutritional value of broccoli seedlings grown at HT on the level of phytochemicals, macro- and microelements, antioxidant capacity, and their extracts’ in vitro cytotoxicity. Total phenols, soluble sugars, carotenoids, quercetin, sinapic, ferulic, *p*-coumaric, and gallic acid were induced by HT. Contrarily, total flavonoids, flavonols, phenolic acids, hydroxycinnamic acids, proteins, glucosinolates, chlorophyll *a* and *b*, and porphyrins were reduced. Minerals As, Co, Cr, Hg, K, Na, Ni, Pb, Se, and Sn increased at HT, while Ca, Cd, Cu, Mg, Mn, and P decreased. ABTS, FRAP, and *β*-carotene bleaching assay showed higher antioxidant potential of seedlings grown at HT, while DPPH showed the opposite. Hepatocellular carcinoma cells were the most sensitive toward broccoli seedling extracts. The significant difference between control and HT-grown broccoli seedling extracts was recorded in mouse embryonal fibroblasts and colorectal carcinoma cells. These results show that the temperature of seedling growth is a critical factor for their nutritional value and the biological effects of their extracts and should definitely be taken into account when growing seedlings for food purposes.

## 1. Introduction

High temperatures caused by global climate change [1] lead to a significant reduction in crop yield, which is an enormous problem because the global population is constantly increasing [2]. High temperature changes the structure of both plant shoot and root systems [3,4], usually in a negative way as far as biomass is concerned. In addition to reducing biomass, there is an even greater challenge because heat stress in plants alters their physiology and biochemistry [5]. It affects phytohormones, structures of membranes, chloroplasts, and mitochondria of different plant species [6,7,8,9]. Additionally, as an adjustment to heat stress, plants produce reactive oxygen species (ROS) [1]. To make it more challenging, in order for each plant species to adapt to environmental conditions as successfully as possible, these changes are often species-specific [5]. Whether the intensity of these changes is so great that it will also significantly change their nutritional value is still not well investigated.

Namely, thus far, from the nutritional point of view, the influence of high temperature has mainly been investigated during the plant material processing, i.e., extraction, drying temperature, cooking, and storage of plant food [10,11,12,13,14,15]. For instance, the total phenolic content and radical-scavenging ability of *Ocimum gratissimum* extracts were significantly dependent on the extraction temperature and solvent type [10]. Higher drying temperature of apricots resulted in a lower decrease of neochlorogenic and chlorogenic acid and catechin, but a higher decrease of epicatechin and quercetin-3-*O*-glucoside, indicating specific effects of a certain temperature toward different compounds [11]. Cooking decreased polyphenols, with great variability between the species emphasizing the importance of the plant matrix [13]. Furthermore, storage temperature is also a factor influencing the total phenolic content and antioxidant capacity of broccoli florets [16]. However, there is less information on the influence of high growing temperature on the nutritional value of plant foods [17]. Since, due to climate change, the temperature is constantly rising, it is necessary to investigate the potential consequences of this effect on the nutritional value of plants.

Another important factor is that the consequences of high temperatures are mainly being investigated in plants at a mature stage. However, even though they are not the predominant developmental stage in a human diet, seedlings are becoming increasingly prevalent in everyday consumption [18]. This is not surprising since it was shown that phenolics and glucosinolates were present in an amount that was ten-fold higher during the first days of germination than in adult plants [19]. Total phenolics in kohlrabi, red cabbage, turnip, rutabaga, and radish sprouts were the highest at the beginning of their 12 days of development [20]. Chinese cabbage seedlings were of higher nutritional quality than plants with two or four real leaves [21]. *Amaranthus* sp. and *Hibiscus sabdariffa* microgreens, compared to field-grown mature foliage, had a lower amount of digestible carbohydrates and Ca but a higher amount of digestible proteins, P, K, Mg, Fe, Mn, and Zn [22]. Likewise, seedlings have higher levels of nutrients and lower contents of antinutrients than ungerminated seeds [23]. From a breeding point of view, they are much less demanding than adult plants, in fact, they don’t even need a substrate, they have a sufficient supply of food in the seed, and only water is sufficient for their cultivation. Another very important fact, contrary to the field-grown crops that can be contaminated, microgreens can be cultivated in controlled environments like greenhouses or phytotrons, which presents one risk management solution for the food safety challenges within the fresh produce industry [24]. All these data contribute to the recognition of seedlings, or microgreens as they are often called, as a crop of modern agriculture [25]. The highest number of different microgreens used in human nutrition can be found in the family Brassicaceae, for example mustards, cabbages, broccoli, cauliflower, radishes, wasabi, arugula, cresses, kohlrabi, turnip, kale, bok choi, collard, nasturtium, Brussels sprouts [26]. However, species from the families Asteraceae (lettuce, endive, sunflower, garland chrysanthemum, marigold), *Apiaceae* (celery, cilantro, fennel, parsley, carrot), *Amaranthaceae* (spinach, amaranth, beets, Swiss chard), *Aliaceae* (chives, scallions, shallots, onions, garlic) are also very common in diets [26]. Due to their delicate tissues, a shelf life of fresh cut microgreens is up to ten days at 5 °C [27]. In spite of and in addition to their high nutritional value, a more intense flavor and taste in comparison with the mature stage of the plant makes this food attractive to the consumer. Additionally, as stated in the work of Zhang et al. [25], since microgreens can easily be grown hydroponically, they are among the most adopted crops of controlled environment agriculture. Given that they are made of very biochemically active and responsive young tissues, the possibilities of adjusting their nutritional potential in a positive sense, i.e., boosting, are great. Thus, more and more information about the possibility of microgreens biofortification appears. Some of the strategies include interspecific transfer of metabolites [28], or addition of minerals into the germination medium [29,30,31]. Alongside pre-harvest interventions, post-harvest treatments such as packaging method, storage temperature, and lighting also significantly affect the nutritional value of microgreens [25]. Besides, many factors influence microgreens nutritional profile, bioactivity, and their consumer acceptance, such as selection of appropriate species, growing systems, substrates, quality of seeds, seeding and germination, irrigation and fertilization, harvesting, phytosanitary quality, and post-harvest storage practices [32]. Due to nutritional benefits, microgreens are even recognized as functional foods in diet-based disease prevention [33]. Moreover, NASA scientists are investigating the possibilities and benefits of growing microgreens in space [34].

Regarding the effect of high growing temperature on seedlings, so far there is very little information. It was shown that increased temperature intensifies root interactions among plant seedlings [4]. Since root structure is crucial for water and nutrient uptake, as well as physical support, its change might significantly affect the seedlings physiological and phytochemical profile and, therefore, nutritional value. For example, very recently it has been revealed that higher growing temperatures in seedlings of wheat increased the activity of antioxidant enzymes catalase, peroxidase, and superoxide dismutase [35].

Due to the wealth of antioxidant phytochemicals and health promoting properties [36], broccoli is among the most consumed vegetables in the world [37]. Numerous in vitro, animal, and human studies show the association of sulforaphane, a biologically active in vivo metabolite of glucosinolate glucoraphanin found in high concentration in broccoli, with induction of mammalian phase II enzymes [38]. Moreover, sulforaphane is recognized as one of the most potent inducers of phase II enzymes [38].

Therefore, the aim of this study was to assess the effect of high growing temperature on the nutritional value of broccoli seedlings in relation to the level of total phenolics, proteins, glucosinolates, sugars, photosynthetic pigments, antioxidant capacity, macro- and microelements, as well as their extracts’ in vitro cytotoxicity and (anti)proliferation ability. For that purpose, we (a) measured the content of different groups of phenolics, sugars, proteins, glucosinolates, chlorophylls, carotenoids, and porphyrins in control and stressed broccoli seedlings, (b) tentatively identified and quantified individual phenolic compounds and vitamin C (*L*-ascorbic acid) using reversed-phase high-performance liquid chromatography (RP-HPLC), (c) quantified macro- and microelements using inductively coupled plasma mass spectrometry (ICP-MS), (d) analyzed antioxidant potential (using ABTS, DPPH, FRAP, and *β*-carotene bleaching assay), (e) examined cytotoxic effects (using MTT and neutral red assays) of extracts of control and stressed seedlings, (f) statistically evaluated the impact of high temperature on the metabolic parameters of broccoli using one way analysis of variance (ANOVA), (g) estimated the relationship between plant groups and measured variables using principal component analysis and hierarchical clustering, and (h) estimated the level of correlations between the measured variables using Pearson’s correlation coefficients to conclude if and how high of a temperature, on the level of above-mentioned parameters, affects broccoli seedlings’ nutritional value.

## 2. Materials and Methods

### 2.1. Materials

*Brassica oleracea* L. convar. *botrytis* (L.) Alef. var. *cymosa* Duch. (broccoli Calabrais) seeds Art. No. 424430 were purchased from International Seeds Processing GmbH (Quedlinburg, Germany), sterilized using 2.55% Izosan aqueous solution for 20 min, and then thoroughly washed with deionized water. After sterilization, in the late autumn and early winter of 2021/2022 seeds were planted in pots containing sterile soil substrate Stender B400 and grown at regular temperature (23 °C day/18 °C night) in the climate chamber FitoClima 600 PLH (Aralab, Rio de Mouro—Portugal) until the seeds sprouted. After sprouting (~7 days), three biological replicas were grown at high temperature (38 °C day/33 °C night) and three biological replicas were maintained at regular temperature (23 °C day/18 °C night) in the climate chamber. In all biological replicas, the light regime was 16 h (day) and 8 h was dark (night), humidity was 65%, and watering was supervised. Plant material was collected 12 days after planting for biological replicas grown at regular temperature, and 20 days after planting for biological replicas grown at high temperature by cutting beneath the lower leaves. Afterwards, plant material was frozen under the stream of liquid nitrogen and lyophilized.

### 2.2. Preparation of Extracts

Lyophilized broccoli seedling tissue was extracted with 70% ethanol for determination of total phenolics, flavonoids, flavonols, hydroxycinnamic acids, total phenolic acids, anthocyanins, soluble sugars, individual phenolics, *L*-ascorbic acid, and antioxidant capacity; with heated 70% ethanol (90 °C) to determine glucosinolates; with 50 mM phosphate buffer pH 7.0 to determine proteins; or with 80% acetone for photosynthetic pigments determination. Extracts at a concentration of 30 mg/mL were used in all methods, except for soluble sugars, those extracts had a concentration of 0.9 mg/mL, extracts for determination of photosynthetic pigments had a concentration of 5 mg/mL, for determination of hydroxycinnamic acids and flavonols a concentration of 6 mg/mL, and for determination of FRAP antioxidant potential a concentration of 15 mg/mL because the values exceeded the upper detection limit. The extracts were prepared as follows: solvent was added to the plant material, shaken in a vortex mixer for 1 min followed by 20 rpm rotation for 60 min at room temperature. The obtained extracts were centrifuged for 5 min at 13,000 rpm and the supernatants were filtered through Whatman SPARTAN syringe filter (Merck KGaA, Darmstadt, Germany), pore size 0.45 μm, and stored at −20 °C until further analysis.

### 2.3. The Amount of Phenolics, Soluble Sugars, Proteins, Glucosinolates, and Pigments

Using a FLUOstar Optima (BMG Labtech GmbH, Ortenberg, Germany), spectrophotometric analyses of non-hydrolyzed extracts were carried out. Total phenolics were measured according to Singleton et al. [39], while flavonoid content was determined as in Zhishen et al. [40]. Measurement of total hydroxycinnamic acids and flavonols was carried out according to Howard et al. [41]. Total phenolic acid content was determined using the European Pharmacopoeia spectrophotometric method [42]. The amount of total anthocynins according to Lee et al. [43], and total glucosinolates content was calculated according to Aghajanzadeh et al. [44]. The amounts of proteins were determined as described in Bradford [45], and soluble sugars according to Dubois et al. [46]. The concentration of photosynthetic pigments was measured according to Sumanta et al. [47] using NanoDrop 2000c (Thermo Fisher Scientific, Waltham, MA, USA).

### 2.4. RP-HPLC Analysis of Individual Phenolics and L-Ascorbic Acid

The extracts were hydrolyzed for two hours at 80 °C and 300 rpm with hydrochloric acid at a final concentration of 1.2 mol/L in order to identify and quantify individual phenolic aglycones and *L*-ascorbic acid. Agilent 1100 Series device with UV/VIS detector was used. The separation was carried out on a Poroshell 120 SB-C18 non-polar column (4.6 × 75 mm, 2.7 μm particle size) using the Zorbax Rx-C18 guard column (4.6 × 12.5 mm, 5 μm particle size). The conditions of separation, identification, and quantification of compounds were as in Šola et al. [21]. Mobile phase A was 0.2% acetic acid (acetic acid:H_2_O; 0.2:99.8; *V/V*), and mobile phase B was 0.2% acetic acid and 80% methanol (acetic acid:MeOH:H_2_O; 0.2:80:19.8; *V/V*). The flow rate was 1 mL/min and the applied volume of each sample was 50 μL. The gradient profile (A/B) was as follows: at 0 min = 100/0, at 42 min = 20/80, at 43 min = 0/100, at 45 min = 0/100, and at 45.1 min = 100/0. The absorbance was measured at 254 nm for *L*-ascorbic acid, 280 nm for gallic acid, 310 nm for chlorogenic, *p*-coumaric, salicylic, caffeic, ferulic, and sinapic acid, and 374 nm for flavonoids quercetin, kaempferol, luteolin, and isorhamnetin. The obtained chromatograms were analyzed using the ChemStation software B.04.03-SP1 [87] (Agilent Technologies, Santa Clara, CA, USA). The compounds were characterized according to their retention times, and the UV spectra were compared with commercial standards. For the quantitative analyses, calibration curves were obtained by injection of known concentrations (within the range 1–250 μg/mL) of the mixed standard solution in triplicate.

### 2.5. Inductively Coupled Plasma Mass Spectrometry Analysis of Macro- and Microelements

Freeze-dried plant material was chopped up with ceramic scissors to obtain homogenous mixture. Each sample (0.12 g) was weighted in triplicate in Teflon vessels and acid-digested in an UltraCLAVE IV microwave digestion system (Milestone, Milan, Italy). Element (arsenic (As), calcium (Ca), cadmium (Cd), cobalt (Co), chromium (Cr), copper (Cu), iron (Fe), mercury (Hg), potassium (K), magnesium (Mg), manganese (Mn), sodium (Na), nickel (Ni), phosphorus (P), lead (Pb), selenium (Se), tin (Sn), thallium (Tl), and zinc (Zn)) quantification was conducted by means of inductively coupled plasma mass spectrometry (ICP-MS; Agilent 7500cx, Agilent Technologies, Tokyo, Japan) according to a previously described method [48]. Purified (duoPUR, Milestone, Milan, Italy) nitric acid (p.a. 65%, Merck, Darmstadt, Germany) and ultrapure water obtained with a GenPure system (TKA, Regensburg, Germany) were used for sample digestion and dilution. For analytical quality control, standard reference material (1570a Spinach and 1573a Tomato leaves, National Institute of Standards and Technology, Gaithersburg, MD, USA) was processed in duplicate with study samples. Method detection limits (MDL) were calculated as the mean concentration plus three times the standard deviation of ten blank samples and presented in Supplementary Materials (Table S1). All results are expressed on a dry mass basis.

### 2.6. Antioxidant Capacity

Free radical scavenging activity (ABTS, DPPH and FRAP) of non-hydrolyzed extracts was measured as described in Šola et al. [21] and bleaching of *β*-carotene was performed according to Chaillou and Nazareno [49] adapted to different volumes. All measurements were performed on a FLUOstar Optima microplate reader. Results are expressed as % of value obtained from Trolox solution of the same concentration as our extracts (i.e., 30 mg/mL for all the methods, except for FRAP which was 15 mg/mL).

### 2.7. In Vitro Cytotoxicity

The in vitro cytotoxicity of broccoli seedling extract was determined on MEF (mouse embryonal fibroblasts), HaCaT (normal human keratinocytes), HepG2 (liver cells—hepatocellular carcinoma), HCT116 (colon cells—colorectal carcinoma), and H460 (lung carcinoma) cell lines using dimethylthiazol diphenyltetrazolium bromide (MTT) and neutral red uptake assay. The sources of the cell lines are as follows: MEF (established in the laboratory of Dr. Pinterić from mice strain RRID:IMSR_JAX:002448), HaCat (RRID:CVCL_0038; gift of Dr. Marijeta Kralj, Ruđer Bošković Institute, Zagreb, Croatia), HepG2 (HB-8065, ATCC, Manassas, VA, USA), HCT116 (CCL-247, ATCC, Manassas, VA, USA), and H460 (HTB-177, ATCC, Manassas, VA, USA).

#### 2.7.1. Dimethylthiazol Diphenyltetrazolium Bromide (MTT) Assay

Cells were cultured as monolayers and maintained high glucose Dulbecco’s modified eagle medium (DMEM, Sigma-Aldrich, St. Louis, MO, USA) with 10% fetal bovine serum (FBS, Sigma-Aldrich, St. Louis, MO, USA), 1% nonessential amino acids (Sigma-Aldrich, St. Louis, MO, USA), and 1% antibiotic/antimycotic solution (Capricorn Scientific, Ebsdorfergrund, Germany) in a humidified atmosphere with 5% CO_2_ at 37 °C. For the assay, 3 × 10^3^ cells were seeded in 96-well plate and 24 h later were treated with different concentrations of the test extracts diluted in growth medium. Working dilutions were freshly prepared on the day of testing (ranging from 0.8 mg/mL to 0.05 mg/mL). Untreated cells were used as a control. In addition, the same dilution of ethanol in growth medium was prepared and incubated with the cells. After 72 h, the treatment was removed, 1 × MTT (Sigma-Aldrich, St. Louis, MO, USA) at a concentration of 20 μg/40 μL was added, and the cells were incubated for 4 h in the growth conditions. After 4 h of incubation the precipitates were dissolved in 160 μL of dimethyl-sulphoxide (DMSO, Gram-mol, Zagreb, Croatia). The absorbance (OD, optical density) was measured on a microplate reader (LabSystem Multiskan MS, Artisan Technology group, Champaign, IL, USA) at 570 nm. The absorbance is directly proportional to the cell viability. The results are expressed as half-maximal (50%) inhibitory concentration (IC50).

#### 2.7.2. Neutral Red Uptake Assay

For this assay, 4 × 10^3^ cells were seeded in 96-well plate and 24 h later were treated with different concentrations of the test extracts diluted in growth medium. Working dilutions were freshly prepared on the day of testing (ranging from 0.8 mg/mL to 0.05 mg/mL). Untreated cells were used as a control. In addition, the same dilution of ethanol in growth medium was prepared and incubated with the cells. After the treatment, the medium with the test substance was removed, and 100 μL of working solution of the neutral red dye (Sigma-Aldrich, St. Louis, MO, USA) was added to each well. The cells were then incubated for 45 min at 37 °C, so that the dye was transported into the cells and accumulated in the lysosomes. The dye was then removed, and the cells were washed twice with 100 μL of PBS buffer each to remove excess dye. After washing, 100 μL of destaining solution, which pulls the dye from the cells, was added to each well. The staining intensity was measured on a microplate reader (LabSystem Multiskan MS, Artisan Technology group, Champaign, IL, USA) at a wavelength of 540 nm, whereby the staining intensity is proportional to cell survival. The results are expressed as the cell viability percentage (%).

### 2.8. Statistical Analysis

Three biological replicas and three technical replicas of each biological replica were utilized in each experiment. The Statistica 14.0 program (TIBCO Software Inc., Palo Alto, CA, USA) was used to statistically analyze the data. The means of the samples were compared using one-way variance analysis (ANOVA) and Duncan’s new multiple range test (DNMRT), a post hoc multiple comparison test. Values that differed at the *p* ≤ 0.05 level were deemed statistically significant. To determine how similar/different samples are based on their phytochemical and antioxidant properties, multivariate principal component analysis (PCA), hierarchical clustering using Euclidean distance between samples, and single linkage clustering were all carried out. The Pearson’s linear correlation coefficients between phytochemical compounds and antioxidant capacity values were computed.

## 3. Results and Discussion

Environmental temperature is an inevitable parameter of plant growth. It affects both above-ground and below-ground parts of a plant organism, causing adaptation processes on several levels, morphological, physiological, and phytochemical [50]. The consequences are species-specific and also depend on the plant’s growth stage. In scope of this work, the effect of high growing temperature on broccoli seedlings was studied in order to reveal the intensity of changes in broccoli phytochemical parameters and their extracts bioactivity. The results contribute to the assessment of global warming impact on the nutritional value of seedlings.

### 3.1. Effect of High Growing Temperature on the Amount of Different Groups of Phenolics in Broccoli Seedlings

Phenolic compounds are specialized metabolites which are one of the most important groups of phytochemicals in plants with protective roles from abiotic and biotic factors [51]. They are synthesized via the phenylpropanoid biosynthesis pathway, typically activated under harmful environmental conditions, leading to the build-up of several phenolic chemicals [52]. In our study, high growing temperature increased total phenolics by 45%, while they decreased flavonoids (−9%), total phenolic acids (−22%), hydroxycinnamic acids (−14%), and flavonols (−23%) (Table 1). Total anthocyanins were resistant to the effect of high temperature. An increase in total phenolics with high temperature has previously been noted in several Brassicaceae species, i.e., cabbage [53] and broccoli sprouts [54]. In contrast, no significant impact of high temperature on phenolic content was observed in kale [53] and rocket sprouts [54], while a heat shock of 45 °C and 50 °C 1 h/day for a week had a negative impact on tomatoes [55]. Furthermore, heat shock also had a negative impact on flavonoid content of tomatoes [55], which was the case in our study as well. We assume that the increase of total phenolics in broccoli seedlings was due to other subgroups of these compounds that we did not measure in scope of this work.

### 3.2. Effect of High Growing Temperature on the Amount of Total Glucosinolates, Proteins, and Soluble Sugars

Glucosinolates are one of the most distinctive phytochemicals in the Brassicaceae family, and their decomposition products function as defense metabolites. Their amount is determined by the organ, developmental stage, and the impact of exogenous factors [56,57]. In our study, we observed a decrease in total glucosinolate content by 40% when seedlings were grown at high temperature (Table 1). Similar to this, Rodríguez et al. [58] found that seedlings of broccoli cultivated at 30 °C had lower total glucosinolate content than seedlings grown at 20 °C. On the other hand, contrary to this, Valente Pereira et al. [59] noticed an increase in total glucosinolate content with high temperature in broccoli sprouts, and a decrease of the content with sprout maturity. However, they used high-performance liquid chromatography to evaluate the concentration of individual glucosinolates, and the total glucosinolate content was estimated by summing up the values of the tested individual glucosinolates, which can explain the difference in findings. Since it was demonstrated that a glucosinolate content decreases with plant maturity [59,60], it is possible that was the reason why, in our experiment, seedlings grown at high temperature had lower total glucosinolate content. Namely, despite being phenotypically in the same stage, they were older than a control group.

Proteins play a crucial role in adjusting physiological traits to altered environments [61]. In our research, protein concentration significantly decreased with high temperature (Table 1). This result suggests avoidance of high temperature when cultivating broccoli seedlings in order to preserve the protein content. Similar results were observed in tobacco [62], lentil [63], strawberry [64] and bentgrass [65]. A possible explanation for lower levels of proteins may be an inhibition of protein synthesis and/or increased denaturation as a result of high temperature [62,65].

Soluble sugars (SS) function as osmoprotectants, they protect a plant from ROS damage, and act as signaling molecules under stressful conditions [66]. An increase of osmolytes during high temperature stress contributes to protein and membrane stability [67]. In our research, soluble sugars increased 216% when seedlings were grown at high temperature (Table 1). This is consistent with studies by Zhang et al. [1], who found that Chinese cabbage of the heat-tolerant ‘268’ line increased soluble sugars at the 8th and 10th day of heat treatment of 40 °C/30 °C (light/dark) and after 4 days of recovery at 25 °C. After 8 days, they also noticed an increase in soluble sugars in the heat-sensitive ‘334’ line; however, this increase eventually started to decline at the 10th day, even though it was still higher than that of the control group. The fact that high temperature increases soluble sugars emphasizes their general role as a plant defense mechanism, and the significance of growing temperature for plant nutritional value.

### 3.3. Effect of High Growing Temperature on Photosynthetic Pigments in Broccoli Seedlings

The amount of chlorophyll in leaf tissue indicates a plant photosynthetic capacity and is influenced by nutrient availability and environmental stresses such as drought, salinity, cold, and heat [68]. Carotenoids, the second-most common naturally occurring pigments in plants, play a role in a number of biological functions in plants, including photosynthesis, photoprotection, and free radical scavenging [62,69]. We observed a decrease in total chlorophyll content (−38%) with high temperature, as well as a decrease in both Chl *a* (−14%) and Chl *b* (−60%) (Table 2). Chl *b* was more sensitive to high temperature, hence Chl *a*/*b* ratio was higher under high temperature (+134%), which was expected given that Chl *b* degrades more rapidly than Chl *a* under stress [70]. Carotenoids were increased (+307%) and Chl/Car ratio was decreased (−84%) in broccoli seedlings grown at high temperature. Similarly, Cui et al. [70] observed a decrease in total chlorophyll content and Chl/Car ratio, and an increase in Chl *a*/*b* and carotenoids in two tall fescue cultivars in response to high temperature. Furthermore, Yang et al. [62] noticed a decrease in total chlorophyll content and an increase in carotenoids in tobacco grown at high temperature. On the other hand, Soengas et al. [53] found that high temperature had a negative impact on Chl *b* in kale and cabbage, but had no significant impact on Chl *a*, indicating a higher susceptibility of Chl *b* to high temperature which we also observed in our survey. We presume that a decline in chlorophyll content results from damage to the photosynthetic apparatus, whereas an increase in carotenoids is a seedling’s strategy to adapt to high temperatures and protect the apparatus.

### 3.4. Effect of High Growing Temperature on Phenolics and Vitamin C in Control and Stressed Broccoli Seedlings

The main antioxidants in Brassicaceae family are phenolic compounds and vitamin C (*L*-ascorbic acid). Their content varies significantly among and within each species and can be influenced by abiotic and biotic factors [52,71]. In our work, *L*-ascorbic acid was not affected (Table 3), indicating the capability of broccoli seedlings to maintain *L*-ascorbic acid homeostasis under high temperature. Similarly, Ragusa et al. [54] observed a non-significant increase in *L*-ascorbic acid concentration at 30 °C in broccoli and rocket sprouts compared with 20 °C. Furthermore, Mahla et al. [72] discovered that the concentration of *L*-ascorbic acid increased in wheat seedlings when exposed to high temperatures, and that this increase was more noticeable in heat-sensitive genotypes than in heat-tolerant genotypes.

High temperature increased 6 out of 11 analyzed individual phenolics (Table 3). The highest increase was in quercetin (+390%) suggesting that this flavonoid has a role in the adaptation of broccoli seedlings to high temperature. This is interesting because quercetin was not a predominant flavonoid in broccoli seedlings. Namely, in control plants, kaempferol was present with almost 12× higher concentration than quercetin, however, it was not significantly changed by high temperature. Since quercetin, compared to kaempferol, has one hydroxyl group more in its B ring, this could be a reason for its higher significance for defense against high temperature. Namely, the antioxidant capacity of phenolics is mainly related to the number of hydroxyl groups, the more they have, the better protection against ROS [52]. Therefore, even though kaempferol is a predominant flavonoid in broccoli seedlings, under a high temperature stress broccoli increases the amount of flavonoid with higher antioxidant potential. Similar to our result, Jan et al. [73] observed an increase in quercetin and kaempferol concentration in rice exposed to combined heat and salt stress and concluded that accumulation of those flavonols increased tolerance. When we checked for the concentration of isorhamnetin, a methylated form of quercetin, it was not significantly changed. Since isorhamnetin, similar to kaempferol, has just one free hydroxyl group on the B ring, it is a weaker antioxidant and obviously plays a less important role in the protection of broccoli seedlings than quercetin. On the other hand, flavanone luteolin has two hydroxyl groups on its B ring and, as we expected, it was significantly (+60%) increased in order to protect the seedlings from high temperature stress. Compared to quercetin, luteolin has no hydroxyl group on the C ring, so its antioxidant potential is lower than that of quercetin [74], and this is probably the reason why the seedlings more significantly increased quercetin concentration for their defense.

Phenolic acids are among the widespread phenolics in Brassica plants [56]. Their content is determined by the species, but also by external factors, as evidenced by our research. Sinapic, ferulic, *p*-coumaric, and gallic acid in broccoli seedlings were increased at high temperature (Table 3). Chlorogenic, salicylic, and caffeic acid were not affected by this type of stress. Similarly, Akin et al. [75] have found out that high temperature led to an accumulation of *p*-coumaric and ferulic acids in tall fescue.

In broccoli seedlings grown under room temperature, the predominant phenolic component was sinapic acid (3.57 ± 0.34 mg/g dw), while in those grown under high temperature that was gallic acid (5.03 ± 0.41 mg/g dw). Out of the identified phenolic acids, ferulic acid was the most susceptible to high temperature with an increase of 168%. Laddomada et al. [76] have found out that under extreme heat stress, minor phenolic acids in wheat increased while the main ones decreased, whereas under extreme drought, the levels of both the main and total phenolic acids increased.

### 3.5. Effect of High Growing Temperature on Macro- and Microelements in Broccoli Seedlings

Heat treatment significantly changed the concentration of 17 elements when compared with the control treatment (Table 4). Ten elements were increased: arsenic (As, +291%), cobalt (Co, +234%), chromium (Cr, +105%), mercury (Hg, +69%), potassium (K, +17%), sodium (Na, +34%), nickel (Ni, +227%), lead (Pb, +170%), selenium (Se, +97%), and tin (Sn, +31%); and seven were decreased: calcium (Ca, −36%), cadmium (Cd, −30%), copper (Cu, −17%), magnesium (Mg, −17%), manganese (Mn, −18%), phosphorus (P, −49%), and thallium (Tl, −57%). Two more elements were decreased (iron and zinc), but not significantly. Similar to this, heat treatment changed element composition in quinoa [77]. In contrast to our findings, Collado-González et al. [78] found that in cauliflower subjected to high temperature, only calcium rose out of the investigated macronutrients (Na, K, Ca, Mg, and P). On the other hand, they discovered that, among micronutrients, copper and manganese were significantly reduced while iron was reduced but not significantly, as was the case in our study.

### 3.6. Effect of High Growing Temperature on Antioxidant Potential of Broccoli Seedlings

To combat stressful conditions, plants developed antioxidant mechanisms which help them reduce ROS. In this study, four different methods, ABTS, DPPH, FRAP, and *β*-carotene bleaching assay were used to determine whether and how did high temperature affect the antioxidant capacity of broccoli seedlings. Based on three out of the four used methods, high temperature significantly increased the antioxidant potential of broccoli seedlings (Table 5). The only method that showed a significant decrease in antioxidant potential with high temperature was DPPH. Such disparities in responses could be attributed to the various chemical mechanisms upon which these methods are based, with ABTS assay being superior for an assessment of antioxidant activity in samples which contain both hydrophilic and lipophilic antioxidants and pigments [79]. Additionally, the color interference of DPPH with samples containing anthocyanins causes the antioxidant activity to be underestimated [80]. Furthermore, given that DPPH reacts slowly with samples, not reaching a stable state even after 8 h [80], it is possible that a 30-min incubation period was insufficient for DPPH to react with antioxidants in broccoli seedlings. Our findings indicate that broccoli seedlings cultivated at high temperature have higher total phenolics (Table 1) and individual phenolics (Table 3), which supports the enhanced antioxidant activity. These outcomes are consistent with the observations made by Soengas et al. [53], who similarly noted greater ABTS values in cabbage and kale cultivated at higher temperatures. We assume that an increase in antioxidant potential is a result of acclimatization to high temperature.

### 3.7. Effect of High Growing Temperature on In Vitro Cytotoxicity of Broccoli Seedling Extract

**Figure 1:**
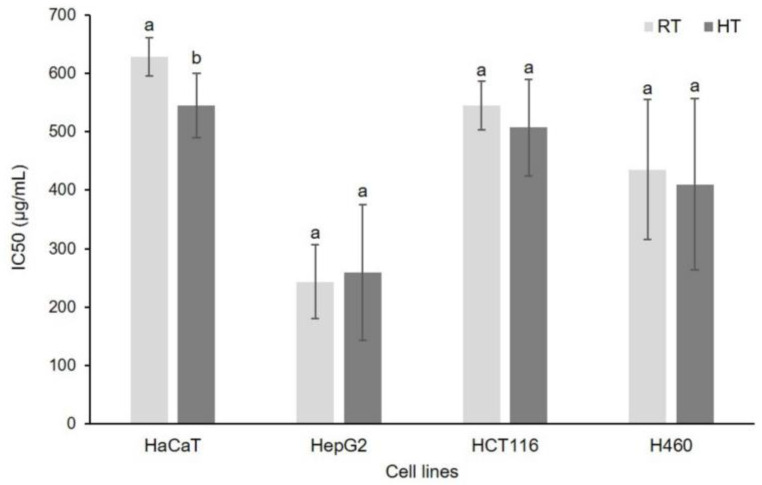
MTT assay showing the viability, expressed in IC50 values, of HaCaT, HepG2, HCT116, and H460 cells after exposure to the extracts of broccoli seedlings grown under room (RT) and high temperature (HT). Values represent mean ± standard deviation of three biological replicates and three technical replicas. Different letters indicate a significant difference among the values for each cell type separately (ANOVA, Duncan’s test, *p* ≤ 0.05). HaCaT = normal human keratinocytes, HepG2 = hepatocellular carcinoma cells, HCT116 = colorectal carcinoma cells, and H460 = lung carcinoma cells.

Cytotoxic potential of broccoli seedling extracts depends on their phytochemical content. The 3-(4,5-dimethylthiazol-2-yl)-2,5-diphenyltetrazoliumbromide (MTT) and neutral red uptake assays are commonly used tests to determine the cytotoxic properties of plant extracts. MTT assay is a quantitative colorimetric assay used for measuring cellular metabolic activity as an indicator of cell viability, proliferation, and cytotoxicity. Since we recorded significant changes of phytochemical profile under the high temperature stress, we assumed that their cytotoxic potential might be changed as well. With that in mind, using MTT assay, we have analyzed IC50 values for five different types of cell cultures, MEF (mouse embryonal fibroblasts), HaCaT (normal human keratinocytes), HepG2 (liver cells—hepatocellular carcinoma), HCT116 (colon cells—colorectal carcinoma), and H460 (lung carcinoma) cell lines (Figure 1). The cytotoxic effect we assessed based on the minimal concentration of extract that is needed for 50% of the cell survival (IC50). Mouse embryonal fibroblasts had IC50 higher than 800 µg/mL, so we concluded that broccoli seedling extract, in reasonable concentrations, do not show cytotoxicity against these cells (data not included in the graph). A similar result was previously recorded for the extracts of three day-old sprouts of two broccoli varieties, *Palam smridhi* and *P. vichitra* [81]. Among the other four cell types, hepatocellular carcinoma cells were the most sensitive toward broccoli seedling extracts, their IC50 values were 243.23 µg/mL and 259.18 µg/mL for control and stressed group, respectively. Obviously, high temperature stress did not significantly affect the cytotoxic potential of broccoli seedlings toward this type of cell. Cytotoxicity against lung and colorectal carcinoma cells was lower than against the hepatocellular cell culture. The IC50 value for control and stressed plant extracts was 435.24 µg/mL and 410.01 µg/mL, respectively, against lung, and 544.67 µg/mL and 507.19 µg/mL, respectively, against colorectal cells. High temperature stress did not affect cytotoxicity of broccoli seedling extracts toward these types of cells. The only type of cells that were differently affected by control and stressed broccoli seedling extracts were normal human keratinocytes. Namely, extract of high temperature-grown seedlings showed significantly higher cytotoxicity against human keratinocytes than the extract from the control group of plants. This suggests that high temperature induces phytochemicals in broccoli seedlings that show a certain level of toxicity toward human keratinocytes. However, based on the IC50 categorization of extracts into four groups, i.e., ≤20 µg/mL—active, >20–100 µg/mL—moderately active, >100–1000 µg/mL—weakly active, and >1000 µg/mL—inactive [82], it should be emphasized that the broccoli seedling extracts belonged to the group of weakly active, which means that their potential harmful effects against these cells are almost negligible. For comparison, very recently Paśko et al. [83] revealed the IC50 of four-day-old broccoli sprout extracts against normal and two types of cancerous thyroid cells, and the values against cancerous cells, after 72 h of incubation as in our experiment, were one order of magnitude lower than in our experiment. Normal cells were not affected, similar to the mouse embryonal fibroblasts in our experiment. This emphasizes plant developmental stage- and cell type-specificity of broccoli extracts’ cytotoxicity.

**Figure 2:**
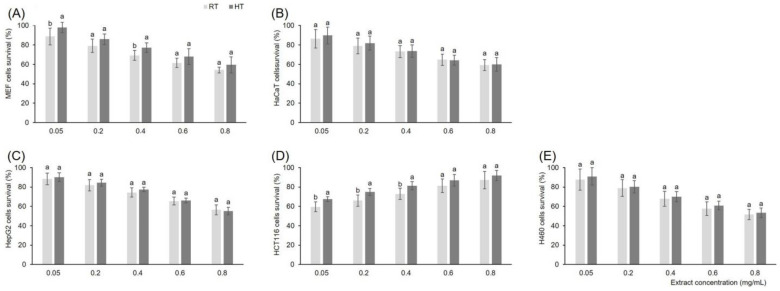
Viability of (**A**) MEF, (**B**) HaCaT, (**C**) HepG2, (**D**) HCT116, and (**E**) H460 cells measured by means of neutral red uptake after incubation with different concentrations of extracts of broccoli seedlings grown under room (RT) and high temperature (HT). Values represent mean ± standard deviation of three biological replicates and three technical replicas. Different letters indicate a significant difference among the values for each extract concentration separately (ANOVA, Duncan’s test, *p* ≤ 0.05). MEF = mouse embryonal fibroblasts, HaCaT = normal human keratinocytes, HepG2 = hepatocellular carcinoma cells, HCT116 = colorectal carcinoma cells, and H460 = lung carcinoma cells.

In addition to the MTT, we also used the neutral red assay to measure the cell viability under the effects of control and stressed plant extracts (Figure 2). Neutral red assay is one of the common methods used to detect cell viability or drug cytotoxicity. The concentrations of extracts were in the range 0.05–0.80 mg/mL and the effects were concentration dependent. In all cell types, the percentage of cell survival decreased with the increase of extract concentration. The significant difference between control and high temperature-grown broccoli seedling extract was recorded in mouse embryonal fibroblasts and colorectal carcinoma cells. Normal human keratinocytes, hepatocellular carcinoma, and lung carcinoma cell lines did not show different survival under the effect of control and stressed broccoli extracts of the same concentration. Mouse embryonal fibroblasts showed significantly higher survival after treatment with high temperature-grown broccoli seedling extract than with the extracts of control plants at the concentrations of 0.05 and 0.40 mg/mL. We recorded the same trend for the colorectal carcinoma cells as well, as cells treated with high temperature-grown broccoli seedling extract had significantly higher survival than with the extracts of control plants at the concentrations of 0.40, 0.60 and 0.80 mg/mL. In the case of embryonal fibroblasts it would be advisable to use extract, at the concentration of 0.05 and 0.40 mg/mL of high temperature-grown broccoli seedling. However, with colorectal carcinoma cells this would be exactly opposite, at extract concentrations of 0.40, 0.60, and 0.80 mg/mL control broccoli seedlings would be preferred over those grown under high temperature. These results show that growing temperature can change the phytochemical profile of broccoli seedlings to the extent that it also changes the in vitro bioactivity of their extracts. Therefore, the temperature of seedling growth is a critical factor not just for their nutritional value, but also for the biological effects of their extracts and should definitely be taken into account when growing seedlings for food purposes.

### 3.8. Statistical Analysis

**Figure 3:**
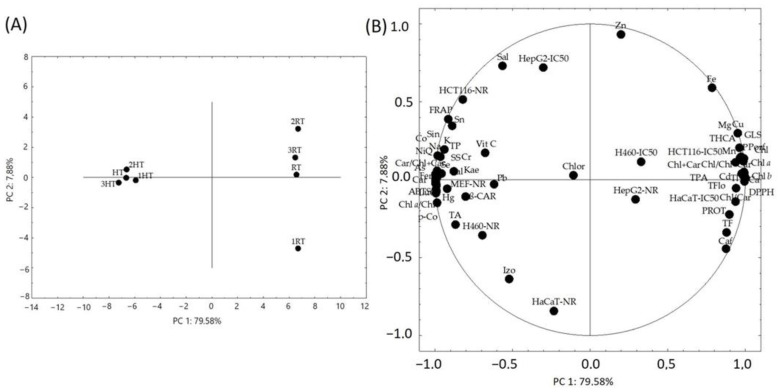
Principal component analysis of the groups of broccoli seedlings: (**A**) score plot separating the samples based on the measured phytochemicals (individual and groups), macro- and microelements, antioxidant capacity, and in vitro cytotoxicity, and (**B**) loading plot of the measured variables.

Loadings for the first two principal components (PC) of the PC analysis (PCA) model showed clear separation of broccoli seedlings grown under room and high temperature (Figure 3A). The first two principal components explained 87.46% of the variance between the measured parameters. Each biological replica and their average value were closer to each other for broccoli grown under high temperature, than for broccoli grown under room temperature. This is interesting because it shows that under high temperature stress the variability, on the level of phytochemicals, antioxidant, and cytotoxic potential, among the broccoli seedling replicas are lower than when grown under room temperature. One of the possible reasons might be the fact that under the high growing temperature, seedlings are generally excited and try to defend themselves against stress with the same mechanisms, which results in their higher similarity. On the other hand, at room temperature, their metabolism is directed to different biosynthetic pathways and mechanisms, which results in their higher variability. Another thing that is apparent is that all the seedling replicates grown under room temperature differed almost exclusively with respect to PC2.

The grouping of the measured variables is shown on the Figure 3B (close up view on the Figure S1A,B). The phytochemical variables that mostly contributed to the separation of high growing temperature broccoli seedlings were quercetin, luteolin, ferulic acid, carotenoids, mass share of carotenoids in photosynthetic pigments, chlorophyll *a*/*b*, cobalt, arsenic, and mercury. Regarding the in vitro bioactivity, the variables that most clearly contributed to the separation of high growing temperature broccoli seedlings were ABTS, FRAP, and *β*-carotene bleaching antioxidant capacity, as well as the viability of mouse embryonal fibroblasts, lung, and colorectal carcinoma cell lines upon treatment with broccoli extracts.

The phytochemical variables that mostly contributed to the separation of broccoli seedlings grown under room temperature were chlorophyll *a*, chlorophyll *b*, total chlorophylls, photosynthetic pigments, total phenolic acids, total flavonols, porphyrins, mass share of chlorophylls in photosynthetic pigments, total glucosinolates, phosphorous, and thallium. The in vitro bioactivity variables that most clearly contributed to the separation of seedlings grown under room temperature were DPPH antioxidant capacity and IC50 values of broccoli extracts toward normal human keratinocytes and colorectal carcinoma cells. The variable that generally had the least effect on the separation of control and stressed seedlings was chlorogenic acid.

**Figure 4:**
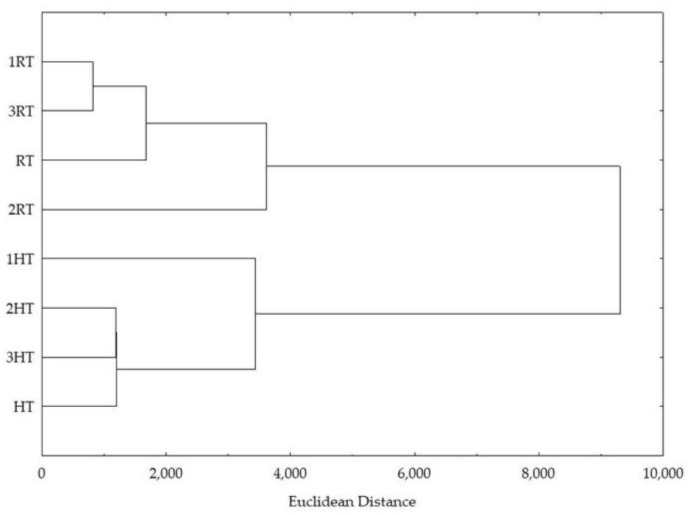
Hierarchical clustering, expressed as Euclidean distance, of control and treated groups of plants, based on the measured total and individual bioactive compounds, their pigments, oxidative stress parameters, antioxidant potential, and ability to inhibit enzymes α-amylase, α-glucosidase, and lipase. HT = plants watered with high temperature water, CT = plants watered with cold water, and Con = plants watered with room temperature water.

Hierarchical clustering confirmed the separation of control and stressed broccoli seedlings (Figure 4). Namely, all the replicas of room temperature grown broccoli seedlings formed one cluster, and all the replicas of high temperature grown broccoli seedlings formed another one.

Pearson’s correlation coefficients revealed very strong negative correlations between phytochemicals kaempferol, quercetin, luteolin, sinapic, ferulic, and *p*-coumaric acid and seedling extracts’ IC50 values toward normal human keratinocytes and colorectal carcinoma cells (Table S2). This suggests that the mentioned phytochemicals most significantly contribute to the cytotoxicity of extracts toward human keratinocytes and colorectal carcinoma cells. These phenolics are also very strongly positively correlated with the viability of mouse embryonal fibroblasts, indicating their importance for fibroblasts survival.

## 4. Conclusions

Due to climate change, high temperature environments are more and more frequent. In order to survive, plants adapt their metabolism, which consequently may affect their nutritional value. Since *Brassica* varieties belong to the most common vegetables in the human diet, and seedlings are more and more common in everyday human life, in this work we have analyzed the effect of high growing temperature on the nutritional value of broccoli seedlings. Results have shown that HT increased 26 of the measured 62 variables, decreased 22 variables, and 14 variables were resistant to this growth condition. The most significant change (+390%) among the individual phytochemicals we recorded for quercetin, which suggests that this flavonol has an important role in the adaptation of broccoli seedlings to HT. HT also significantly increased total phenolics, soluble sugars, carotenoids, sinapic, ferulic, *p*-coumaric, and gallic acid, the minerals potassium and sodium, and antioxidant capacity measured by ABTS, FRAP, and *β*-carotene bleaching assay. *L*-ascorbic acid was not affected, indicating the capability of broccoli seedlings to maintain vitamin C homeostasis under high temperature. Among the tested cell cultures, hepatocellular carcinoma cells were the most sensitive toward broccoli seedling extracts. The only type of cells that were differently affected in MTT assay by control and stressed broccoli seedling extracts were normal human keratinocytes. Neutral red assay revealed the significant difference between control and high temperature-grown broccoli seedling extracts in mouse embryonal fibroblasts and colorectal carcinoma cells. These results show that growing temperature can change the phytochemical profile of broccoli seedlings to the extent that it also changes the in vitro bioactivity of their extracts. Therefore, the temperature of seedling growth is critical for their nutritional value and biological effects of their extracts and should definitely be taken into account when growing seedlings for food purposes.

## Figures and Tables

**Table 1:** Effect of high growing temperature on phytochemical content of broccoli seedlings.

	Room Temperature	High Temperature	Δ (%)
TP (mg GAE/g DW)	17.53 ± 2.04 ^b^	25.49 ± 1.51 ^a^	+45
TF (mg QE/g DW)	20.91 ± 0.98 ^a^	19.12 ± 3.63 ^b^	−9
TFlo (mg QE/g DW)	10.65 ± 3.22 ^a^	8.15 ± 1.61 ^b^	−23
THCA (mg CAE/g DW)	11.86 ± 2.11 ^a^	10.21 ± 1.67 ^b^	−14
TPA (mg CAE/g DW)	7.59 ± 0.57 ^a^	5.90 ± 0.95 ^b^	−22
TA (mg C-gluE/g DW)	11.60 ± 5.90 ^a^	16.40 ± 5.92 ^a^	+41
GLS (mg SINE/g DW)	88.91 ± 6.24 ^a^	53.72 ± 7.78 ^b^	−40
PROT (mg BSAE/g DW)	57.56 ± 7.19 ^a^	51.86 ± 5.98 ^b^	−10
SS (mg SUCE/g DW)	9.41 ± 7.69 ^b^	29.75 ± 6.05 ^a^	+216

Values represent mean ± standard deviation of three biological replicates and three or more technical replicas. Different letters indicate a significant difference among the values in a row (ANOVA, Duncan’s test, *p* ≤ 0.05). TP = total phenolics; TF = total flavonoids; TFlo = total flavonols; THCA = total hydroxycinnamic acids; TPA = total phenolic acids; TA = total anthocyanins; GLS = total glucosinolates; PROT = total proteins; SS = total soluble sugars; GAE = gallic acid equivalents; QE = quercetin equivalents; CAE = caffeic acid equivalents; C-gluE = cyanidin-3-glucoside equivalents; SINE = sinigrin equivalents; BSAE = bovine serum albumin equivalents; SUCE = sucrose equivalents.

**Table 2:** Effect of high growing temperature on photosynthetic pigments content in broccoli seedlings.

mg/g DW	Room Temperature	High Temperature	Δ (%)
Chl *a*	5.07 ± 0.34 ^a^	4.38 ± 0.55 ^b^	−14
Chl *b*	5.88 ± 0.45 ^a^	2.36 ± 0.76 ^b^	−60
Chl *a* + Chl *b*	10.94 ± 0.46 ^a^	6.74 ± 1.30 ^b^	−38
Chl *a* + Chl *b* + Car	11.12 ± 0.41 ^a^	7.69 ± 1.27 ^b^	−31
Chl *a*/*b*	0.86 ± 0.09 ^b^	2.01 ± 0.41 ^a^	+134
Chl/(Chl + Car)	0.98 ± 0.02 ^a^	0.87 ± 0.03 ^b^	−11
Car/(Chl + Car)	0.02 ± 0.01 ^b^	0.13 ± 0.03 ^a^	+539
Chl/Car	43.32 ± 12.39 ^a^	7.11 ± 1.61 ^b^	−84
Car	0.23 ± 0.10 ^b^	0.96 ± 0.05 ^a^	+307
Por	21.95 ± 0.96 ^a^	13.00 ± 2.56 ^b^	−41

Values represent mean ± standard deviation of three biological replicates and three technical replicas. Different letters indicate a significant difference among the values in a row (ANOVA, Duncan’s test, *p* ≤ 0.05). Chl = chlorophyll; Car = carotenoids; Por = porphyrins.

**Table 3:** Effect of high growing temperature on the concentration of *L*-ascorbic acid and individual phenolic components in broccoli seedlings.

mg/g DW	Room Temperature	High Temperature	Δ (%)
*L*-ascorbic acid	1.32 ± 0.03 ^a^	1.41 ± 0.12 ^a^	+6
Kaempferol	2.13 ± 0.60 ^a^	2.48 ± 1.10 ^a^	+16
Luteolin	0.39 ± 0.01 ^b^	0.06 ± 0.00 ^a^	+60
Isorhamnetin	0.06 ± 0.03 ^a^	0.06 ± 0.01 ^a^	+6
Quercetin	0.18 ± 0.11 ^b^	0.82 ± 0.25 ^a^	+390
* Total identified flavonoids *	2.40 ± 0.68 ^a^	3.43 ± 1.35 ^a^	+43
Sinapic acid	3.57 ± 0.34 ^b^	4.25 ± 0.14 ^a^	+19
Ferulic acid	0.55 ± 0.10 ^b^	1.46 ± 0.15 ^a^	+168
Chlorogenic acid	0.10 ± 0.02 ^a^	0.10 ± 0.02 ^a^	0
*p*-Coumaric acid	0.25 ± 0.05 ^b^	0.35 ± 0.10 ^a^	+42
Gallic acid	2.61 ± 0.93 ^b^	5.03 ± 0.41 ^a^	+93
Salicylic acid	0.03 ± 0.00 ^a^	0.03 ± 0.00 ^a^	+8
Caffeic acid	0.15 ± 0.05 ^a^	0.12 ± 0.04 ^a^	−20
*Total identified phenolic acids*	7.14 ± 0.84 ^b^	11.34 ± 0.34 ^a^	+57
*Total identified phenolics*	9.64 ± 1.52 ^b^	14.77 ± 1.57 ^a^	+53

Values represent mean ± standard deviation of three biological replicates and three technical replicas. Different letters indicate a significant difference among the values in a row (ANOVA, Duncan’s test, *p* ≤ 0.05).

**Table 4:** Effect of high growing temperature on the concentration of macro- and microelements in broccoli seedlings.

	Room Temperature	High Temperature	Δ (%)
As (µg/kg DW)	201.31 ± 4.42 ^b^	786.70 ± 6.56 ^a^	+291
Ca (g/kg DW)	16.59 ± 0.26 ^a^	10.65 ± 0.47 ^b^	−36
Cd (µg/kg DW)	122.63 ± 4.57 ^a^	86.39 ± 4.71 ^b^	−30
Co (µg/kg DW)	59.29 ± 4.72 ^b^	197.76 ± 3.12 ^a^	+234
Cr (µg/kg DW)	97.25 ± 13.99 ^b^	199.01 ± 47.42 ^a^	+105
Cu (mg/kg DW)	7.07 ± 0.32 ^a^	5.86 ± 0.01 ^b^	−17
Fe (mg/kg DW)	145.78 ± 13.61 ^a^	124.46 ± 1.66 ^a^	−15
Hg (µg/kg DW)	14.41 ± 0.87 ^b^	24.31 ± 0.92 ^a^	+69
K (g/kg DW)	67.10 ± 2.51 ^b^	78.22 ± 2.41 ^a^	+17
Mg (g/kg DW)	5.45 ± 0.18 ^a^	4.52 ± 0.11 ^b^	−17
Mn (mg/kg DW)	49.30 ± 1.22 ^a^	40.59 ± 0.98 ^b^	−18
Na (g/kg DW)	10.81 ± 0.35 ^b^	14.52 ± 0.30 ^a^	+34
Ni (µg/kg DW)	249.95 ± 14.24 ^b^	818.15 ± 50.39 ^a^	+227
P (g/kg DW)	6.19 ± 0.36 ^a^	3.13 ± 0.07 ^b^	−49
Pb (µg/kg DW)	51.08 ± 7.42 ^b^	138.08 ± 97.95 ^a^	+170
Se (µg/kg DW)	50.39 ± 2.88 ^b^	99.21 ± 7.31 ^a^	+97
Sn (µg/kg DW)	94.58 ± 11.47 ^b^	123.43 ± 5.67 ^a^	+31
Tl (µg/kg DW)	766.41 ± 42.20 ^a^	326.03 ± 4.54 ^b^	−57
Zn (mg/kg DW)	54.70 ± 2.48 ^a^	54.01 ± 0.73 ^a^	−1

Values represent mean ± standard deviation of three biological replicates and three technical replicas. Different letters indicate a significant difference among the values in a row (ANOVA, Duncan’s test, *p* ≤ 0.05).

**Table 5:** Effect of high growing temperature on antioxidant capacity of broccoli seedlings.

Inhibition %	Room Temperature	High Temperature	Δ (%)
ABTS	57.18 ± 5.18 ^b^	93.64 ± 2.24 ^a^	+64
DPPH	88.23 ± 0.87 ^a^	58.41 ± 3.19 ^b^	−34
FRAP	97.74 ± 0.56 ^b^	99.00 ± 0.14 ^a^	+1
*β*-carotene	7.55 ± 3.34 ^b^	13.07 ± 4.85 ^a^	+73

Values represent mean ± standard deviation of three biological replicates and three technical replicas. Different letters indicate a significant difference among the values in a row (ANOVA, Duncan’s test, *p* ≤ 0.05).

## Data Availability

Data is contained within the article.

## Supplementary Materials

The following supporting information can be downloaded at: https://www.mdpi.com/article/10.3390/foods12030582/s1, Figure S1: Principal component analysis of broccoli seedlings: close-up view at the loading plots of the measured variables grouped (A) on the left side of the biplot, and (B) on the right side of the biplot. Table S1: Method detection limits (MDL) and results of the element quantification in standard reference materials used for quality control. Table S2. Pearson’s correlation coefficient (*r*) between the individual and groups of phytochemicals, antioxidant capacity, and in vitro cytotoxicity of broccoli seedling extracts.
