# Supplementary material for: High Growing Temperature Changes Nutritional Value of Broccoli (Brassica oleracea L. convar. botrytis (L.) Alef. var. cymosa Duch.) Seedlings
Source: Foods. 2023 Jan 29;12(3):582. doi: 10.3390/foods12030582 (PMC9914779; doi:10.3390/foods12030582)
Supplement: Supplementary file 1 [file foods-12-00582-s001.zip › Table S1.pdf]

Table S1. Method detection limits (MDL) and results of the element quantification in standard reference materials used for quality control.

Element		MDL*	**NIST SRM 1570a Spinach Leaves**		**NIST SRM 1573a Tomato Leaves**	
			Certified (mean ± uncertainty)	Measured (mean ± SD)	Certified (mean ± uncertainty)	Measured (mean ± SD)
As	µg/kg	3.26	68 ± 12	67 ± 5	112.6 ± 2.4	111.0 ± 4.9
Ca	mg/kg	7.87	15260 ± 660	15177 ± 654	50500 ± 900	50187 ± 758
Cd	µg/kg	0.375	2876 ± 58	2836 ± 71	1517 ± 27	1520 ± 93
Co	µg/kg	0.916	393 ± 30	398 ± 7	577.3 ± 7.1	566.9 ± 26.1
Cr	µg/kg	6.67	/	1671 ± 125	1990 ± 60	1935 ± 46
Cu	mg/kg	0.116	12220 ± 860	12250 ± 890	4700 ± 140	4620 ± 100
Fe	mg/kg	1.27	/	272 ± 15	368 ± 7	367 ± 2
Hg	µg/kg	2.70	29.7 ± 2.1	28.8 ± 0.4	34.1 ± 1.5	34.8 ± 4.3
K	mg/kg	37.12	29000 ± 260	29723 ± 500	27000 ± 500	27794 ± 783
Mg	mg/kg	2.81	9000	8877 ± 31	12000	12062 ± 207
Mn	mg/kg	0.007	76.0 ± 1.2	73.8 ± 3.3	246 ± 8	246 ± 4
Na	mg/kg	3.35	18210 ± 230	18631 ± 724	136 ± 4	138 ± 2.0
Ni	µg/kg	5.01	2142 ± 58	2181 ± 69	1582 ± 41	1527 ± 71
P	mg/kg	12.26	5190 ± 67	5359 ± 83	2161 ± 28	2204 ± 88
Pb	µg/kg	1.399	200[b]	202 ± 7	/	557 ± 45
Se	µg/kg	5.5	115.2 ± 4	112.5 ± 2.8	54 ± 3	54.8 ± 3.8
Sn	µg/kg	2.34	/	27.6 ± 1.3	/	48.4 ± 7.3
Tl	µg/kg	0.453	/	21.0 ± 1.0	/	37.9 ± 2.6
Zn	mg/kg	0.067	82.3 ± 3.9	83.2 ± 3.1	30.9 ± 0.7	31.9 ± 0.9

*Calculated as mean plus three times of the blanks' standard deviation multiplied by dilution factor information value.
