# Supplementary figures and images for: High Growing Temperature Changes Nutritional Value of Broccoli (Brassica oleracea L. convar. botrytis (L.) Alef. var. cymosa Duch.) Seedlings
Source: Foods. 2023 Jan 29;12(3):582. doi: 10.3390/foods12030582 (PMC9914779; doi:10.3390/foods12030582)

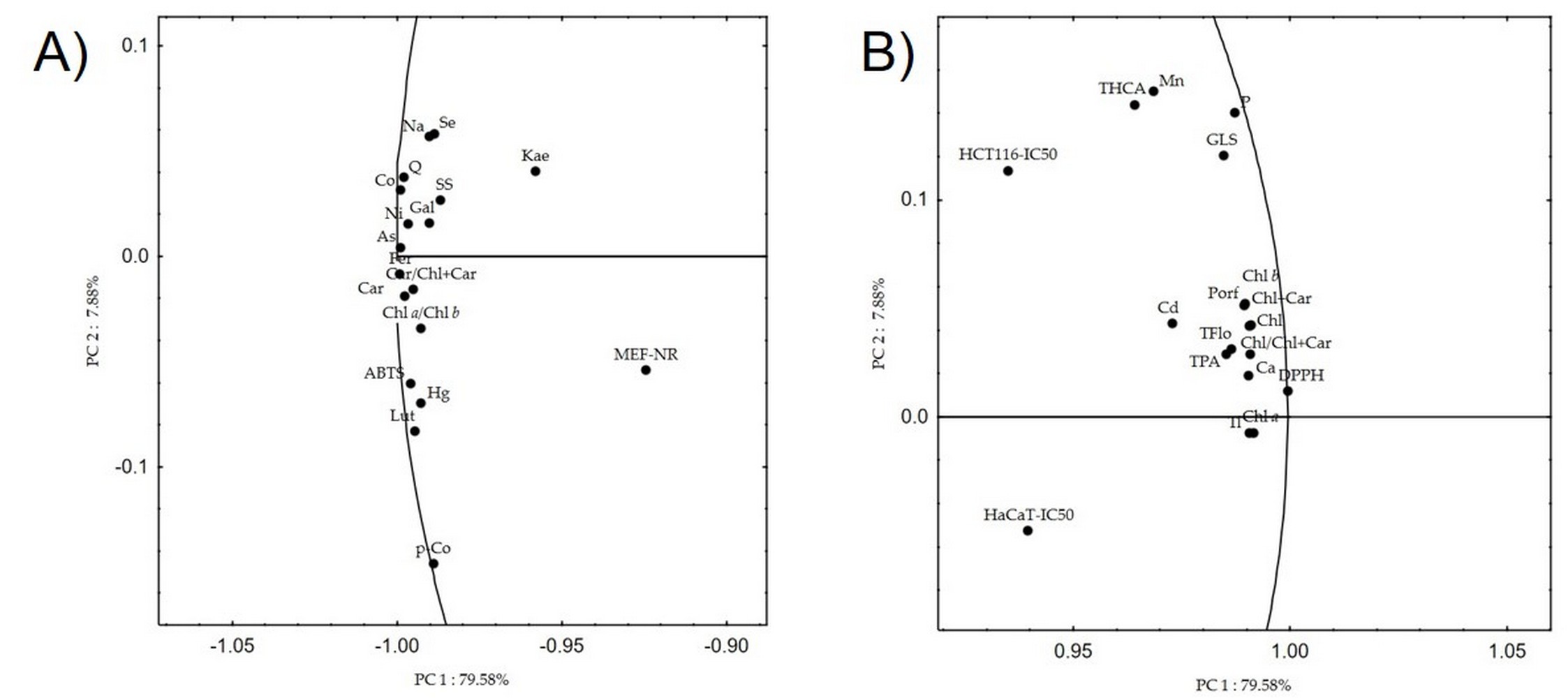

Supplement: Supplementary file 1 [file foods-12-00582-s001.zip › Figure S1.jpg]
